# *Capsicum baccatum* Red Pepper Prevents Cardiometabolic Risk in Rats Fed with an Ultra-Processed Diet

**DOI:** 10.3390/metabo13030385

**Published:** 2023-03-05

**Authors:** Aline Rigon Zimmer, Bianca Franco Leonardi, Eduardo Rigon Zimmer, Alexandre Pastoris Muller, Grace Gosmann, Luis Valmor Cruz Portela

**Affiliations:** 1 Pharmaceutical Sciences Graduate Program (PPGCF), Faculty of Pharmacy, Federal University of Rio Grande do Sul (UFRGS), Porto Alegre 90610-000, RS, Brazil; 2 Graduate Program in Biological Sciences: Pharmacology and Therapeutics (PPGFT), Department of Pharmacology, Federal University of Rio Grande do Sul (UFRGS), Porto Alegre 90035-003, RS, Brazil; 3 Graduate Program in Biological Science: Biochemistry (PPGBioq), Department of Biochemistry, Federal University of Rio Grande do Sul (UFRGS), Porto Alegre 90035-003, RS, Brazil; 4 Department of Biochemistry, Federal University of Santa Catarina (UFSC), Florianópolis 88037-000, SC, Brazil

**Keywords:** *Capsicum baccatum*, cardiovascular risk, dyslipidemia, hyperglycemia, metabolic syndrome

## Abstract

Metabolic syndrome is a serious health condition reaching epidemic proportions worldwide and is closely linked to an increased risk of cardiovascular problems. The lack of appropriate treatment paves the way for developing new therapeutic agents as a high priority in the current research. In this study, we evaluated the protective effects of *Capsicum baccatum* red pepper on metabolic syndrome scenarios induced by an ultra-processed diet in rats. After four months, the ultra-processed diet increased central obesity, triglycerides, total cholesterol, LDL-cholesterol plasma levels, and impaired glucose tolerance. The oral administration of *C. baccatum* concomitantly with the ultra-processed diet avoided the accumulation of adipose tissue in the visceral region, reduced the total cholesterol and LDL fraction, and improved glucose homeostasis, factors commonly associated with metabolic syndrome. The data presented herein reveal an important preventive action of *C. baccatum* in developing metabolic disorders among animals fed a hypercaloric diet, significantly reducing their cardiometabolic risk. Allied with the absence of toxic effects after chronic use, our study suggests *C. baccatum* red pepper as a secure and enriched source of bioactive compounds promising to protect against pathological processes associated with metabolic syndrome.

## 1. Introduction

Metabolic syndrome is a complex disorder represented by a set of factors commonly associated with insulin resistance, central obesity, glucose intolerance, hypertension, hypertriglyceridemia, and low levels of HDL-cholesterol [1]. The association of metabolic disorders such as the increase in plasma cholesterol and the imbalance in glucose homeostasis may lead to the development of metabolic syndrome, which has been reaching a broad segment of the adult population [2]. Enormous interest has arisen in the scientific community for that clinical condition, since it is characterized by a number of factors or conditions at risk of cardiovascular disease [3]. Metabolic syndrome increases the risk of cardiovascular disease by five-times, increasing the overall mortality rate 1.6 times [4].

Several factors contribute to the development of metabolic disorders such as the consumption of ultra-processed diets rich in sugar and fat as well as a sedentary lifestyle. As a consequence, an increase in obesity and the emergence of pathologies such as type 2 diabetes, dyslipidemia, and metabolic syndrome has been observed [5,6,7].

A common alternative frequently used by patients suffering from chronic conditions associated with metabolic disorders refers to the use of natural products with popular description of glycemic and cholesterol control and anti-obesity effects. In the literature, several reports mention plant-based dietary interventions for controlling diabetes and dyslipidemia, and some of them have demonstrated effectiveness in preclinical and clinical trials [8,9,10,11,12].

The genus *Capsicum*, from the Solanaceae family, consists of several species of peppers and have shown interesting therapeutic potential in this context. Various studies conducted with *Capsicum* species, in particular concerning the species *C. annuum* and *C. frutescens*, have demonstrated analgesic and anti-inflammatory activities as well as beneficial properties on glucose and lipid metabolism [13,14,15,16,17,18,19,20,21,22]. The species of *Capsicum baccatum* var. *pendulum* (pimenta dedo-de-moça), one of the most consumed peppers in Brazil, demonstrates potential as an antioxidant and anti-inflammatory agent [23,24,25]. Recently, after a bioguided assay, our group showed that the butanol (BUT) extract of *C. baccatum* presented anti-inflammatory and antioxidant properties correlated with the flavonoid and total phenolic contents [23]. Furthermore, no adverse effects were observed on behavioral, hematological, and metabolic parameters after long-term oral administration of *C. baccatum*, suggesting a level of pharmacological safety [26].

The biological potential and safety of *C. baccatum* BUT extract open the perspective to a deeper understanding of its effects in chronic disorders involving inflammatory pathways and oxidative burden. The inflammatory and redox status are important components associated with chronic diseases that include most forms of cardiovascular disease, type 2 diabetes, and metabolic syndrome, representing the greatest health threats [3,27,28,29].

Continuing the pharmacological investigation of *C. baccatum*, our study aimed to evaluate the impact of BUT extract administration in a metabolic syndrome scenario induced by an ultra-processed highly palatable (HP) diet in rats. This kind of diet, rich in saturated fat and sucrose, produces a state of obesity, dyslipidemia, and glucose intolerance, mimicking several risk factors that contribute to the development of the metabolic syndrome. Thereby, Wistar rats were submitted to 4 months of an ultra-processed HP diet and received BUT extract orally during the diet period. The *C. baccatum* BUT extract was selected due to its appreciable anti-inflammatory, and antioxidant activities previously reported [23]. It is important to emphasize the lack of studies regarding the use of this plant species in metabolic syndrome and the possible prevention of cardiovascular risk factors.

## 2. Materials and Methods

### 2.1. Plant Material and Extraction

*Capsicum baccatum* var. *pendulum* (Willd.) Eshbaugh (Solanaceae) fruit was obtained from a cultivated area in Turuçu, Rio Grande do Sul, Brazil, after being allowed access to genetic resources of the Brazilian Genetic Patrimony Management Council (CGEN number 010393/2015-3). A voucher specimen (ICN 181469) was identified and deposited at the Herbarium of the Universidade Federal of Rio Grande do Sul (UFRGS). The fruit of red pepper was dried in a circulating air stove (40 °C) and triturated to powder. The fruit was submitted to successive extractions in a Soxhlet apparatus in order to obtain the butanol (BUT) dried extract, enriched in bioactive substances (187.51 mg of total phenolic compounds and 54.68 mg of flavonoids per gram of dried extract), as previously described [23].

### 2.2. Animals and Experimental Design

Adult 60 days Wistar male rats (*Rattus norvegicus*), weighing 200–250 g, were obtained from the Central Animal House of the Department of Biochemistry (UFRGS). The animals were maintained under controlled temperature (22 ± 2 °C) and humidity (55 ± 10%) conditions on a 12 h light-dark cycle (7:00 a.m. and 7:00 p.m.). Animal care followed the international standards for animal protection and official governmental guidelines and was approved by the Ethical Committee on animal use of UFRGS, Brazil (approval number 19446).

The rats were randomly assigned to one of four experimental groups: (1) the standard diet (SD) group (*n* = 10), which received standard laboratory rat chow (50% carbohydrate from starch, 25% protein, and 4% fat) and saline daily by gavage for 130 days; (2) the SD group plus BUT extract (SD + BUT, *n* = 10), which received standard laboratory rat chow and 200 mg/kg of *C. baccatum* BUT extract daily by gavage for 130 days; (3) the ultra-processed highly palatable (HP) diet group (*n* = 10), which received an enriched diet in simple sugars and saturated fat (65% carbohydrates (34% from condensed milk, 8% from sucrose, and 23% from starch), 25% protein, and 10% of fat (soybean oil) and saline daily by gavage for 130 days; and (4) the HP diet group plus BUT extract (HP + BUT, *n* = 10), which received HP diet and 200 mg/kg of *C. baccatum* BUT extract daily by gavage for 130 days [30]. The dosage of 200 mg/kg of *C. baccatum* was selected considering our previous study showed relevant anti-inflammatory and antioxidant properties [23,26]. In addition, the 130 days (nearly 18 weeks) of diet exposure was established according to previous studies of our research group and collaborators, suggesting 13 to 19 weeks of diet consumption to significantly impact the metabolic outcomes in the animals [30,31,32].

All animals had free access to food and water. The animals’ body weight was measured weekly throughout the study period. The initial and final body weight and the weight gain were compared among groups. Clinical signs of toxicity, general appearance, and mortality were monitored daily during the experimental period.

### 2.3. Blood Sampling, Organs, and Tissues Collections

At the end of the experimental protocol, the animals were anesthetized (ketamine:xylazine, 100:10 mg/kg, i.p.) for blood sampling for hematological and biochemical analysis. After blood collection, animals were humanely sacrificed (exsanguinated), quickly dissected, and the liver, brain, heart, and kidneys were excised and weighed individually. Fat tissues from the retroperitoneal and epididymal regions were dissected and weighed as previously described [33], and the total fat mass was considered as the sum of both. Organ/tissue absolute weight was compared with the final body weight of each rat on the day of sacrifice to determine the relative organ/tissue weight (absolute organ/tissue weight (g) × 100/animal body weight (g).

### 2.4. Glucose Tolerance Test and Glycosylated Hemoglobin

A glucose tolerance test was performed 130 days after the beginning of animal experimentation. A 50% glucose solution was injected to 8 h fasted rats (i.p, 2 g/kg body weight), and the blood sample was collected by a small puncture on the tail immediately before, 30, 60, and 120 min after the injection [34]. At each time, glucose was measured by a glucosimeter (AccuChek Active, Roche Diagnostics^®^, São Paulo, SP, Brazil). In addition, glycosylated hemoglobin was determined by ELISA kits (Katal Biotecnológica^®^ Ind. Co., Ltd., Belo Horizonte, MG, Brazil, https://www.katal.com.br/Reagentes (accessed on 2 March 2023) at the end of treatment.

### 2.5. Lipid Profile and Atherogenic Index

The lipid profile of the animals that received the different diets (SD or HP), treated or not with the *C. baccatum* BUT extract, was evaluated through the measurement of the total content of triglycerides (TG), total cholesterol (TC), and the fractions of high-density (HDL-c) and low-density (LDL-c) lipoprotein cholesterol with commercial kits (Katal Biotecnológica^®^ Ind. Co., Ltd., Belo Horizonte, MG, Brazil, https://www.katal.com.br/Reagentes (accessed on 2 March 2023). The atherogenic index (AI), which is the measure of the atherosclerotic lesion extent based on serum lipids, was determined in all groups as (TC)/(HDL-c) [35].

### 2.6. Biochemical and Hematological Analysis

For the assessment of renal and hepatic functions, the plasma levels of creatinine, urea, albumin, alanine aminotransferase (ALT), alkaline phosphatase (ALP), and gamma-glutamyl transpeptidase (GGT) were measured through commercial kits (Katal Biotecnológica^®^ Ind. Co., Ltd., Belo Horizonte, MG, Brazil, https://www.katal.com.br/Reagentes (accessed on 2 March 2023). All analyses were performed at Spectramax^®^ M5 (Molecular Devices, San Jose, CA, USA). Furthermore, the following hematological parameters were determined by using a semiautomatic blood analyzer (MS4, USA): hemoglobin (Hb), red blood cell (RBC) count, hematocrit (HCT), mean corpuscular volume (MCV), mean corpuscular hemoglobin (MCH), mean corpuscular hemoglobin concentration (MCHC), red cell distribution width (RDW), white blood cell (WBC) count, the cytological differential (percentage of lymphocytes and neutrophils), and platelets.

### 2.7. Behavioral Tasks

The effects of the *C. baccatum* BUT extract on spontaneous locomotion, exploratory activities as well as the anxiety-like behavior of the animals were evaluated by the open field task [36], the light–dark exploration task [37], and the elevated plus-maze task [38]. After each trial, the apparatus was cleaned with an ethanol solution (70%). All behavioral tasks were performed between 1:00 PM and 5:00 PM. A video camera positioned above the apparatus was used to record all experimental sessions, and the analysis were performed using a computer-operated tracking system (ANY-maze^®^, Stoelting, Woods Dale, IL, USA).

#### 2.7.1. Open Field Task

The open field task is a widely used model for the evaluation of spontaneous locomotion and exploratory activities. The rats were gently placed in the center of the arena (50 × 50 cm; 50-cm-high walls), and the total distance travelled, and mean speed were measured for 10 min [36].

#### 2.7.2. Light–Dark Exploration Task

The rats were submitted to the light–dark task as described by Crawley and Goodwin [37]. The task consisted of a box (40 × 50 × 60 cm) divided equally into two compartments: the light compartment (60 W light), and the dark compartment (room illumination at 20 W). Rodents are nocturnal animals, preferring darker areas, and the decrease in the exploratory activity in the light area is taken as a measure of anxiety. Animals were gently placed in the corner of the light compartment and left free to explore for 5 min. The following parameters were analyzed: (1) the total time spent in the light compartment; (2) the number of transitions from light to dark, defined as placing the four paws into the light compartment; and (3) the risk assessment behavior index (RA, i.e., the number of investigations of the light compartment by placing some but not all paws).

#### 2.7.3. Elevated Plus-Maze Task

The elevated plus-maze measures anxiety-like behavior in rodents and was performed as previously described [38]. The experiments were conducted under a dim red light in a quiet room. The animals were placed individually on the central platform (5 × 5 cm) of the plus-maze facing one of the open arms and recorded with a video camera for 5 min. The time spent in the open (50 × 10 cm) and closed arms (50 × 10 × 40 cm), the number of entries into the arms, the total distance travelled, and the mean speed were analyzed.

### 2.8. Statistical Analysis

Statistical analysis was performed using GraphPad Prism^®^ 8.0. The results were expressed as the mean ± standard error of the mean (S.E.M.) and statistically analyzed by two-way analysis of variance (ANOVA), followed by a Tukey’s test for multiple comparisons. Differences were considered significant at *p* < 0.05.

## 3. Results

### 3.1. Body Weight, Adipose Tissue Weights

**Figure 1:**
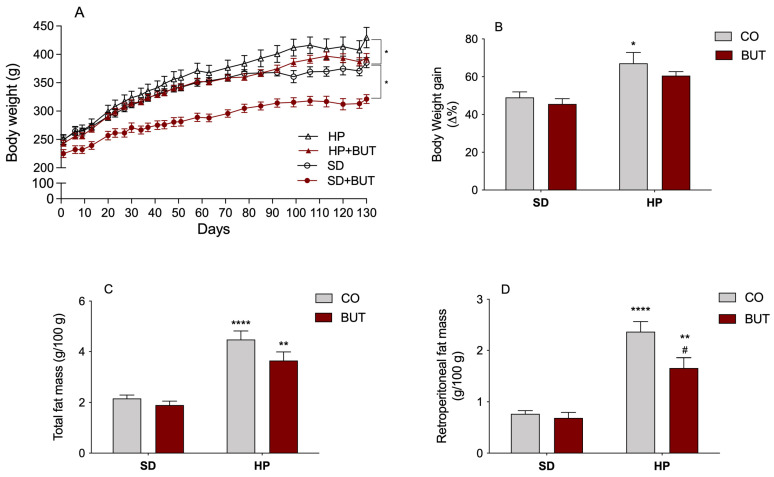
Effect of the *C. baccatum* extract (200 mg/kg, p.o.): (**A**) Growth curves of rats orally treated with *C. baccatum* BUT extract and different diets for 130 days; (**B**) Percentage of body weight gain in different experimental groups at the end of treatment; (**C**) Relative weight (g/100 g body weight) of the total fat mass; and (**D**) relative weight (g/100 g body weight) of the retroperitoneal fat mass after 130 days of treatment with different diets. The results are presented as the mean ± S.E.M. (*n* = 10) after analysis by two-way ANOVA. * *p* < 0.05, ** *p* < 0.01, and **** *p* < 0.001 compared with the standard diet group (SD); # *p* < 0.05 compared with the ultra-processed highly palatable diet group (HP). CO: control group (saline); BUT: 200 mg/kg of *C. baccatum* BUT extract (gavage).

The body weight was monitored throughout the entire trial period (Figure 1A,B) to evaluate the effects of the HP diet intake and the use of the BUT extract of *C. baccatum* on the animal’s body composition. At the end of the experiment, animals receiving the HP diet had significantly higher weight gain (67% increase) than the animals that received the SD diet (49% increase), demonstrating that the HP diet induced an obesity profile (Figure 1B). Evaluating the effects of the BUT extract on body weight, we can verify a reduction in the final weight of the group receiving the SD diet plus BUT extract, despite no differences in the weight gain compared to the control group (SD) (Figure 1B). Furthermore, the group receiving the BUT extract and HP diet (HP+BUT) showed no significant differences in the final body weight and weight gain when compared with the control group (SD), indicating that treatment with the *C. baccatum* extract exhibited a downward in the weight gain over time (Figure 1A,B).

When evaluating the fat tissue deposits, specifically in response to different diets, a substantial increase in the total (107.2%) and retroperitoneal (209.0%) fat pad weight in rats consuming the HP diet was observed compared to the control group (Figure 1C). Notably, the treatment with the BUT extract significantly avoided the accumulation of adipose tissue in the abdominal region of the rats fed a HP diet (Figure 1D), suggesting that there were significant physiological adjustments in regulatory pathways of lipid metabolism, promoting lower triglyceride storage in visceral tissues. Overall, these data highlight the prophylactical positive effect *of C. baccatum* red pepper on the body weight gain and central obesity of rats submitted to an ultra-processed diet, indicating an anti-obesity potential.

### 3.2. Lipid and Glycemic Profiles

**Figure 2:**
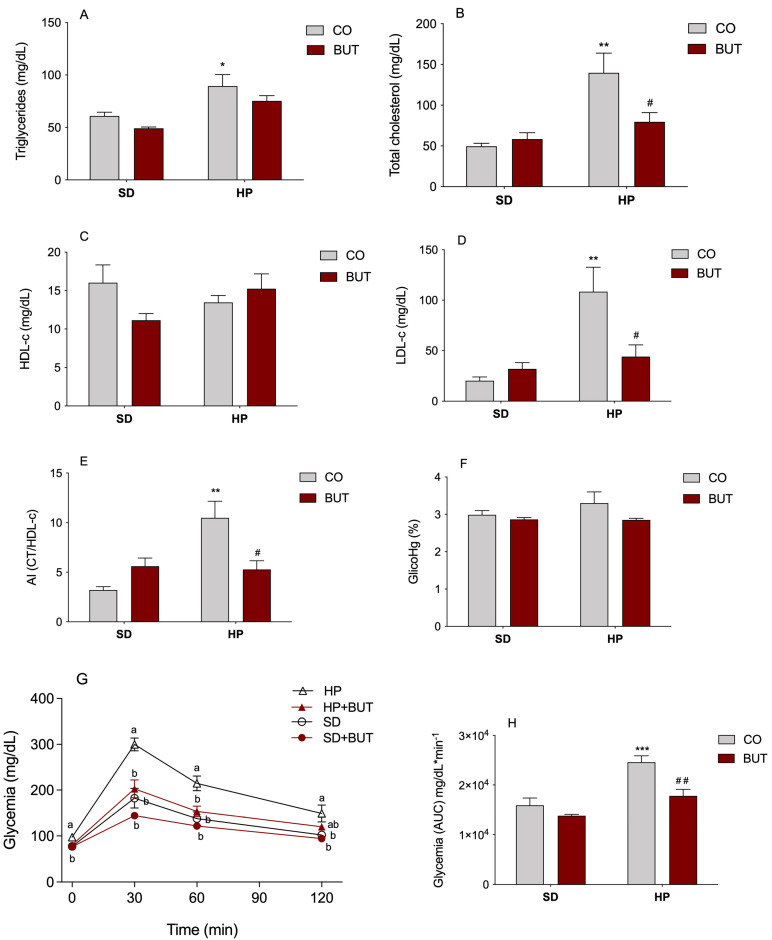
Effect of *C. baccatum* BUT extract (200 mg/kg, p.o.) in the plasma levels of: (**A**) triglycerides (mg/dL); (**B**) total cholesterol (mg/dL); (**C**) HDL-cholesterol (mg/dL); (**D**) LDL-cholesterol (mg/dL); (**E**) atherogenic index (AI) expressed as total cholesterol (CT)/HDL-cholesterol (HDL-c); (**F**) glycosylated hemoglobin (GlicoHg%); (**G**) glucose tolerance test (GGT); and (**H**) area under the curve (AUC) resulting from the GGT test after 130 days of experimentation. The results are presented as the mean ± S.E.M. (*n* = 10) after analysis by two-way ANOVA. For the GGT, repeated-measures analysis of variance (ANOVA) were used to evaluate the statistical significance, different letters are significantly different at *p* < 0.05. *p < 0.05, ** *p* < 0.01, and *** *p* < 0.001 compared with the standard diet group (SD); # *p* < 0.05; and ## *p* < 0.01 compared with the ultra-processed highly palatable diet group (HP). CO: control group (saline); BUT: 200 mg/kg of *C. baccatum* BUT extract (gavage).

The lipid profile of animals treated or not with the BUT extract that received a SD or HP diet is shown in Figure 2A–E. It was verified that there was a significant increase in the content of triglycerides (46.8% increase, Figure 2A), total cholesterol (182.3% increase, Figure 2B), and LDL-cholesterol levels (534.0% increase, Figure 2D) in animals receiving the HP diet compared to animals that received the SD diet. The treatment with the BUT extract (200 mg/kg, per os) for 130 days was able to prevent the increase in the total content of cholesterol (Figure 2B) and LDL-c fraction (Figure 2D) in animals receiving the HP diet (HP + BUT), avoiding the rise in these two parameters in a condition of high intake of carbohydrates and lipids. There was no difference in the plasma HDL-cholesterol among the experimental groups (Figure 2C).

In the determination of the AI (Figure 2E), a widely used parameter for estimating cardiovascular risk, a marked increase in the risk (3.3 times) was observed among the animals that received only the HP diet. On the other hand, the treatment with *C. baccatum* provided a significant reduction in AI compared to the HP group, reducing around two times the risk of cardiovascular events to which these animals were submitted.

The plasma glucose profile of animals receiving both diets, treated or not with the *C. baccatum* BUT extract, was assessed through the glucose tolerance test (GTT). The HP diet triggered significant changes in glucose homeostasis since an impairment in glucose tolerance was evident after 130 days of diet consumption (Figure 2G,H). After this period, the plasma glucose of the animals of the HP group was higher at all time points of the GGT (fasting, 30, 60, and 120 min). The most interesting result is that animals treated with the BUT extract did not develop this glucose intolerance in the presence of the HP diet, maintaining glucose levels in this group (HP + BUT), similar to those observed in the control animals (SD group, Figure 2G). The total AUC of the plasma glucose levels resulting from GGT remained approximately 35% and 30% lower in SD and HP+BUT, respectively, compared to the HP group (Figure 2H). These findings suggest that the *C. baccatum* extract reduced the blood glucose levels and improved glucose tolerance. When the glycosylated hemoglobin of animals was measured, there were no significant differences (Figure 2F).

### 3.3. Examination of the Organs, Biochemical, and Hematological Analysis

All animals appeared healthy at the end of the experimental period. No clinical signs of toxicity including hair loss, piloerection, changes in skin, eyes, or oral mucosa, nor death were observed (Supplementary Figure S1). Visceral examinations of the brain, heart, kidney, and liver of the control and treated rats revealed no visible lesions. Moreover, in Table 1, no significant alterations in the relative weight of the organs were observed among groups (*p* > 0.05).

In order to determine the impact of the different diets and *C. baccatum* BUT extract on the kidney and liver function, some biochemical markers were analyzed (Table 1). There was no significant difference among groups in ALT, ALP, GGT, albumin, urea, and creatinine at the end of the experiment compared to the control group (SD). In the hematological evaluation, there were no significant differences among groups in the analyzed parameters: RBC count, Hb, HCT, MCV, MCH, MCHC, RDW, platelets, WBC count, and lymphocyte and neutrophil content (Table 1).

### 3.4. Behavioral Tasks

**Figure 3:**
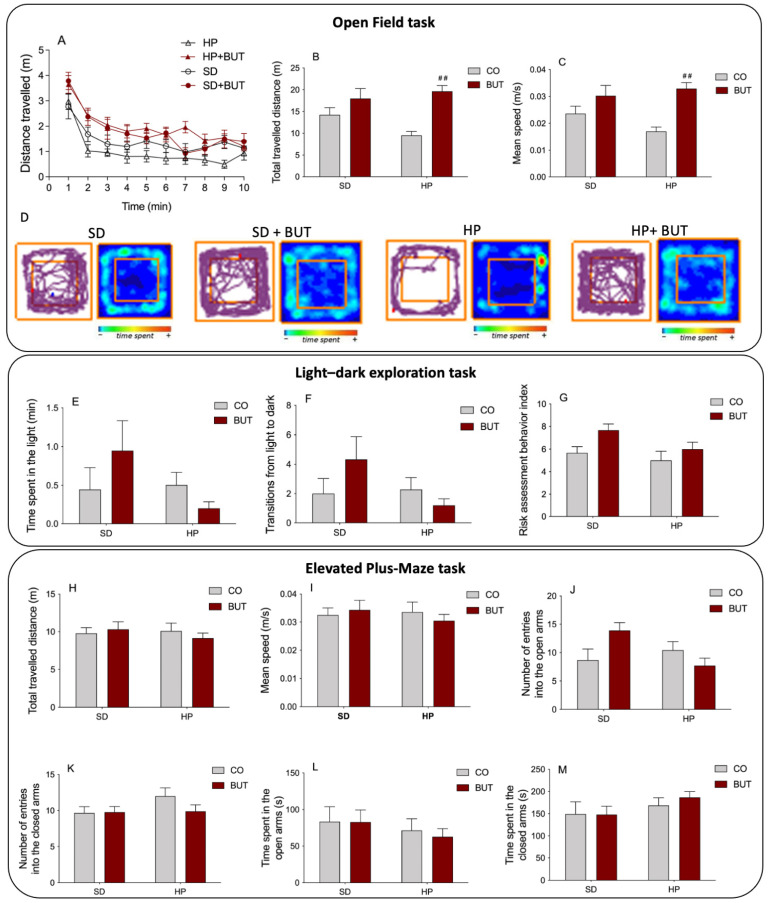
Behavioral assessment performed after 130 days of oral administration of *C. baccatum* BUT extract (200 mg/kg) with different diets. Open field task: (**A**) distance travelled by the animals throughout the experiment minute by minute; (**B**) total distance traveled (m); (**C**) mean speed (m/s); (**D**) representative occupancy plots obtained by video-tracking software (ANY-maze^®^, Stoelting Co., Wood Dale, IL, USA). Light–dark exploration task: (**E**) total time spent in the light compartment (min); (**F**) number of transitions from light to dark; (**G**) risk assessment behavior index. Elevated plus-maze task: (**H**) total travelled distance (m); (**I**) mean speed (m/s); (**J**) number of entries into the open arms; (**K**) number of entries into the closed arms; (**L**) time spent in the open arms (s); (**M**) time spent in the closed arms (s). The results are presented as the mean ± S.E.M. (*n* = 10) after analysis by two-way ANOVA. ## *p* < 0.01 compared with the ultra-processed highly palatable diet group (HP). CO: control group (saline); BUT: 200 mg/kg of *C. baccatum* BUT extract (gavage).

In the behavioral assessment of the different groups, a decrease in exploratory activity and spontaneous locomotion was observed in animals that received the HP diet since they showed a decrease in the total distance travelled (Figure 3A–B) and mean speed (Figure 3C) during the open field task. Figure 3A,D show the analysis of the total distance traveled by the animals throughout the experiment minute by minute, and the degree of occupation of the box, respectively. These data clearly demonstrate the sedentary behavior of the animals receiving the HP diet. Interestingly, the treatment with 200 mg/kg BUT extract reversed this effect of the HP diet, normalizing the exploratory activity and spontaneous locomotion of the animals (HP + BUT).

Regarding the anxiogenic profile of animals, no changes were observed in the light–dark exploration task (Figure 3E–G) and in the elevated plus-maze task (Figure 3H–M). In the light–dark exploration task, there was no difference in the total time spent in the light compartment (Figure 3E), the number of transitions from light to dark (Figure 3F), and the risk assessment behavior index (Figure 3G) compared to the control group. Similarly, in the elevated plus-maze task, no differences were observed among groups in the total distance travelled (Figure 3H, and Supplementary Figure S2), mean speed (Figure 3I), entries in open (Figure 3J) and closed arms (Figure 3K), and time spent in open (Figure 3L) and closed arms (Figure 3M). These results indicate that the BUT extract did not cause alterations in the anxiety-like behavior of the animals.

## 4. Discussion

Diet has been recognized as one of the most critical factors in managing metabolic disorders such as dyslipidemia, diabetes mellitus, and metabolic syndrome [6,39]. An ultra-processed high palatable diet (HP), also named the cafeteria diet, or Western-style diet, is enriched in simple sugars and saturated fat, and its consumption contributes to weight gain, obesity, and insulin resistance [2,7].

Many authors have reported the potential of some spices such as garlic, onion, and peppers to influence the body metabolism by experimentally documented investigations [12,13,40,41]. Red peppers are common in worldwide gastronomy and have been extensively consumed for centuries. The specie *Capsicum baccatum* var. *pendulum* is the most consumed species in Brazil, and concentrations of total phenols and flavonoids were significantly greater in the *C. baccatum* fruit compared with other species of peppers more broadly studied such as *C. annuum* or *C. frutescens* [23,24,42]. A recent chemical characterization of *C. baccatum* fruit identified 42 phenolic substances, and the flavonoids quercetin 3-O-rhamnoside, luteolin 7-O-glycoside, and naringenin were the most abundant compounds described [43].

These polyphenolic compounds are related to positive influences on lipid and glycemic metabolism [44,45,46,47], beyond presenting important antioxidant and anti-inflammatory activities [24,48]. Hence, a large prospective cohort investigated the dietary intake of individual polyphenols and their association with 5-year body weight change. Interestingly, the strongest inverse association with body weight was observed for quercetin 3-O-rhamnoside, suggesting that it may play a protective role against obesity with adipose tissue and systemic oxidative stress as a possible therapeutic target [49]. In addition, naringin has shown beneficial health effects in animals and humans including improved lipid and glucose metabolism and ameliorated cardiovascular dysfunctions [50,51].

Previously, our research group demonstrated that the BUT extract of *C. baccatum* showed the highest antioxidant and anti-inflammatory activities, and the total phenolic and flavonoid contents were positively correlated with both effects [23]. Furthermore, we demonstrated that 60 days of oral administration of the BUT extract had no toxic effects on hematologic, metabolic, and behavioral outcomes in normal male CF1 mice [26]. However, despite our findings, the physiological and pharmacological potential of *C. baccatum* has been scarcely explored.

Based on these results, this study was designed to evaluate the effects of the *Capsicum baccatum* BUT extract on the lipid and glycemic metabolism of animals in conditions of unfavorable diet, mimicking a metabolic syndrome scenario, looking for an agent that associates activities that could be potentially promising for protection against cardiovascular risk factors.

In this concern, the rats received different diets concomitantly with 200 mg/kg of the *C. baccatum* BUT extract or saline solution by gavage for 130 days. At the beginning of the experimental period, the mean body weights of the animals in all groups were very similar. However, at the end of the protocol, the mean body weight of rats fed with the HF diet was significantly greater than those receiving the SD diet. Interestingly, the administration of the *C. baccatum* extract associated with the HF diet prevented excessive weight gain, despite a similar dietary intake among the rats in the study.

The animals’ lipid profile assessment after 130 days found that the treatment of *C. baccatum* BUT extract exerted beneficial effects by preventing the increase in serum total cholesterol and LDL-c fraction, thereby avoiding the rise in the atherogenic index (AI), even when the diet remained high in saturated fats and sugar. The classic lipid profile of the metabolic syndrome is characterized by elevated triglycerides, LDL-c, and low HDL-c, conditions that add to other components to determine a high cardiovascular risk [1].

The value and safety of lowering plasma LDL-c and AI in treating cardiovascular disease have been established unequivocally. In its native and oxidized forms, LDL-c causes direct endothelial cell injury and dysfunction, predisposing to an inflammatory response in the artery wall that promotes the development of an atherosclerotic plaque. Clinical studies have shown that decreasing plasma LDL-c significantly reduces coronary heart disease morbidity and mortality. The same occurs with AI. The higher the AI, the bigger the risk of fatty infiltration in the heart, coronaries, liver, and kidney, promoting oxidative damage to these organs [52,53].

Regarding the glycemic profile of the animals, the BUT extract demonstrated a significant antihyperglycemic ability since, in the presence of high carbohydrate consumption, was able to avoid the emergence of a profile of glucose intolerance, keeping the blood glucose levels of the animals similar to the control group. Insulin resistance and changes in glucose transporters are frequently present in metabolic syndrome. The excess of circulating free fatty acids, which originate from adipose tissue and triglyceride-rich lipoproteins, is an important contributing factor to this frame of insulin resistance. In the liver, free fatty acids increase the production of glucose, triglycerides, and low-density lipoproteins (LDL and VLDL). In the muscle, it reduces insulin sensitivity, inhibiting the uptake of glucose insulin-mediated. All of these factors influence the loss of glycemic control [54].

Another essential parameter in the relationship between metabolism and health concerns body fat distribution. The overweight associated with abdominal fat accumulation (visceral or central obesity) has emerged as a significant risk factor for cardiovascular disease and an essential component in diagnosing metabolic syndrome. Contemporary studies suggest that the accumulation of adipose tissue in the abdominal region precedes the development of other components of this syndrome [55,56]. The visceral adipose tissue is the fat deposit with higher atherogenic potential. On this site, the adipocytes have intense lipolytic activity, releasing large amounts of free fatty acids in the systemic circulation, increasing the endogenous synthesis of lipoproteins [57,58]. The treatment with the *C. baccatum* BUT extract significantly reduced the central obesity of the rats fed the HP diet. This finding, associated with the anti-dyslipidemic effects and the improvement in glucose homeostasis, corroborates the reduction in cardiometabolic risk in the presence of the *C. baccatum* BUT extract.

Upon the need to establish the safety of the chronic use of the BUT extract in rats as well as ensure that the observed effects on the metabolism of are not related to toxicological changes, a behavioral, biochemical, and hematological evaluation was conducted. Furthermore, it is clearly important to perform studies to predict the safety profile of plant species using well-defined methodologies, simulating population use, and preconizing the cumulative toxic effects over long-term administration, as achieved in this research.

Regarding the biochemical and hematological evaluation, no significant alterations that provided evidence of undesirable effects for the *C. baccatum* BUT extract were observed. Furthermore, no signs of toxicity induced by the BUT extract were observed in the hematopoietic system. A normal macroscopic appearance of all organs, particularly for the kidney and liver, the absence of toxicity was confirmed by the serum biochemical markers of the function of these organs. 

Behavioral assessment was performed through the open field, the light–dark, and the elevated plus-maze tasks. No differences were observed among the groups in the light–dark, and elevated plus-maze tasks, evidencing that the *C. baccatum* BUT extract does not induce anxiety-like behavior in the animals. In the open field task, there was a prejudice in the locomotor function of animals receiving the HP diet, confirmed by the reduction in total distance travelled and the mean speed during the test. These alterations may reflect an obese phenotype developed by the animals fed the HP diet once the final body weight of these animals was significantly higher. However, the group that received the HP diet and the *C. baccatum* BUT extract did not show this sedentary-like behavior presented by the HP group. Beyond differences in body weight, lipid, and glycemic profiles, the HP group animals showed a higher abdominal fat pad content than animals fed with the same diet but treated with the BUT extract. This set of factors might have contributed to the animals receiving *C. baccatum* but did not demonstrate alterations in exploratory activities and spontaneous locomotion. Hence, the results of the evaluation of the chronic use of *C. baccatum* in rats did not show any apparent toxic effect on the analyzed parameters.

## 5. Conclusions

The data presented herein suggest that consuming an ultra-processed highly palatable (HP) diet leads to a phenotype of central obesity, unfavorable lipid profile, impaired glucose tolerance, and significantly increased cardiovascular risk. On the other hand, the administration of the *C. baccatum* BUT extract to rats associated with the HP diet prevented the increase in the total cholesterol and LDL-c levels, the imbalance in glucose homeostasis, and the visceral obesity, significantly reducing the cardiometabolic threat. Furthermore, in the safety assessment of *C. baccatum* chronic use, no alterations were observed in the animals’ behavioral, hematological, and biochemical parameters, indicating the absence of toxicity after long-term use.

These results open the venue to developing a secure and bioactive enriched product from *C. baccatum* as a promising multi-target agent preventing metabolic syndrome and the emergence of cardiovascular problems.

## Figures and Tables

**Table 1:** Effects of different diets and the *C. baccatum* BUT extract (200 mg/kg, per os) on the relative weight of visceral organs (g/100 g b.w.), the serum biochemical parameters, and the hematological profile after a 130-day study in rats.

Parameters	SD	SD + BUT	HP	HP + BUT
Brain (g/100 g b.w.)	0.523 ± 0.021	0.586 ± 0.068	0.485 ± 0.050	0.517 ± 0.032
Heart (g/100 g b.w.)	0.291 ± 0.019	0.311 ± 0.027	0.284 ± 0.018	0.274 ± 0.024
Kidney (g/100 g b.w.)	0.645 ± 0.061	0.665 ± 0.055	0.627 ± 0.064	0.569 ± 0.064
Liver (g/100 g b.w.)	2.998 ± 0.134	3.080 ± 0.173	2.670 ± 0.233	2.795 ± 0.288
ALT (IU/L)	21.1 ± 2.3	23.2 ± 11.1	21.3 ± 16.8	14.7 ± 9.8
ALP (IU/L)	133.3 ± 35.8	123.9 ± 39.7	110.8 ± 13.9	98.1 ± 49.1
GGT (IU/L)	4.8 ± 2.1	6.0 ± 2.0	5.3 ± 2.0	9.4 ± 8.4
Albumin (g/dL)	4.0 ± 0.3	4.0 ± 0.2	4.0 ± 0.1	4.2 ± 0.3
Urea (mg/dL)	36.9 ± 5.6	35.8 ± 8.5	33.1 ± 8.0	43.6 ± 12.2
Creatinine (mg/dL)	0.37 ± 0.1	0.35 ± 0.1	0.51 ± 0.1	0.33 ± 0.1
RBC count (10^6^/µL)	6.4 ± 0.8	6.5 ± 0.9	5.8 ± 0.7	6.7 ± 0.7
Hb (g/dL)	14.2 ± 0.9	13.9 ± 0.5	13.4 ± 0.6	13.6 ± 0.4
HCT (%)	33.6 ± 4.8	34.2 ± 5.1	31.5 ± 3.1	35.0 ± 3.9
MCV (µm^3^)	52.7 ± 1.5	52.6 ± 1.0	54.8 ± 1.3	52.5 ± 0.8
MCH (pg)	22.6 ± 2.2	21.8 ± 2.5	23.3 ± 2.1	20.6 ± 2.4
MCHC (g/dl)	42.6 ± 4.7	41.4 ± 5.1	42.7 ± 3.1	39.2 ± 4.6
RDW (%)	13.2 ± 0.3	12.9 ± 0.5	13.6 ± 0.7	12.9 ± 0.5
Platelets (10^3^/mm^3^)	670.5 ± 326.4	732.7 ± 170.4	693.8 ± 107.3	775.0 ± 108.0
WBC count (10^3^/µL)	7.6 ± 2.5	5.9 ± 0.7	6.37 ± 1.4	6.07 ± 1.7
Lymphocytes (%)	77.5 ± 4.7	80.2 ± 4.5	77.5 ± 6.0	79.0 ± 4.6
Neutrophils (%)	16.5 ± 5.6	16.2 ± 4.1	16.0 ± 5.9	14.2 ± 3.9

ALT = alanine aminotransferase; ALP = alkaline phosphatase; GGT = gamma-glutamyltranspeptidase; RBC = red blood cells, Hb = hemoglobin, HCT = hematocrit; MCV = mean corpuscular volume; MCH = mean corpuscular hemoglobin; MCHC = mean corpuscular hemoglobin concentration; RDW = red cell distribution width; WBC = white blood cell. Values are the means ± S.E.M. No significant differences were observed after two-way ANOVA.

## Data Availability

The data presented in this study are available in the main article and the supplementary materials.

## Supplementary Materials

The following supporting information can be downloaded at: https://www.mdpi.com/article/10.3390/metabo13030385/s1. Figure S1: Animals appeared at the end of the experimental period. No clinical signs of toxicity, including hair loss, piloerection, changes in skin, eyes, or oral mucosa, nor death were observed. Figure S2: Elevated plus-maze task: Representative occupancy plots obtained by video-tracking software (ANY-maze^®^, Stoelting Co., USA) for experimental groups after 130 days of oral administration of *C. baccatum* BUT extract (200 mg/kg) with different diets.
