# Supplementary material for: Capsicum baccatum Red Pepper Prevents Cardiometabolic Risk in Rats Fed with an Ultra-Processed Diet
Source: Metabolites. 2023 Mar 5;13(3):385. doi: 10.3390/metabo13030385 (PMC10052057; doi:10.3390/metabo13030385)
Supplement: Supplementary file 1 [file metabolites-13-00385-s001.zip › metabolites-2251421-supplementary.pdf]

Supplementary data

Figure S1 – Animals appeared at the end of the experimental period. No clinical signs of toxicity, including hair loss, piloerection, changes in skin, eyes, or oral mucosa, nor death were observed. SD: control group (standard diet); SD+BUT: standard diet + 200 mg/kg of *C. baccatum* BUT extract; HP: ultra-processed highly palatable diet group (HP); HP+BUT: HP diet + 200 mg/kg of *C. baccatum* BUT extract.

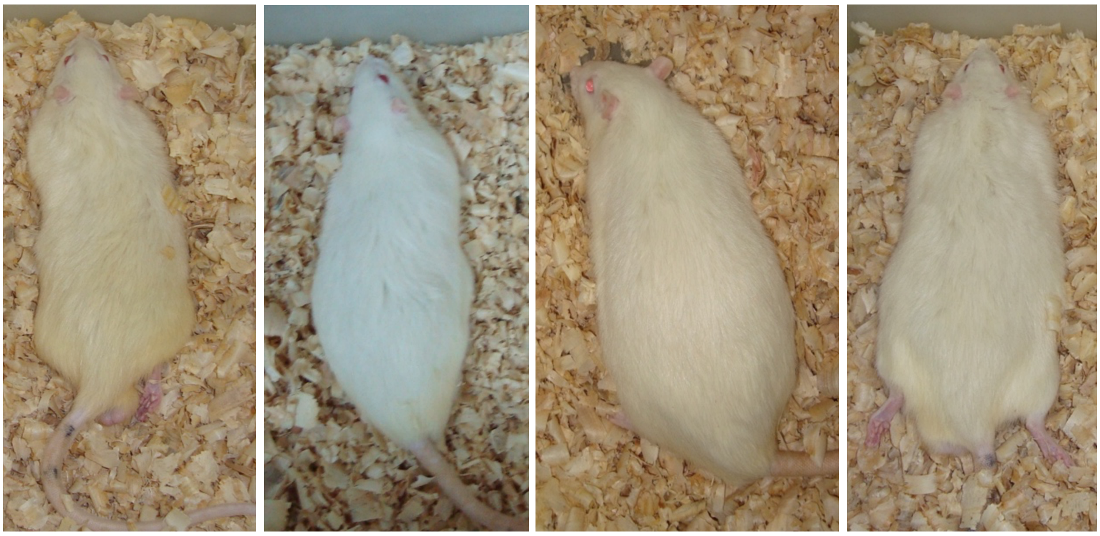

SD SD+BUT HP HP+BUT

Figure S2 - Elevated plus-maze task**:** Representative occupancy plots obtained by video-tracking software (ANY-maze®, Stoelting CO, USA) for experimental groups after 130 days of oral administration of *C. baccatum* BUT extract (200 mg/kg) with different diets. SD: control group (standard diet); SD+BUT: standard diet + 200 mg/kg of *C. baccatum* BUT extract; HP: ultra-processed highly palatable diet group (HP); HP+BUT: HP diet + 200 mg/kg of *C. baccatum* BUT extract.

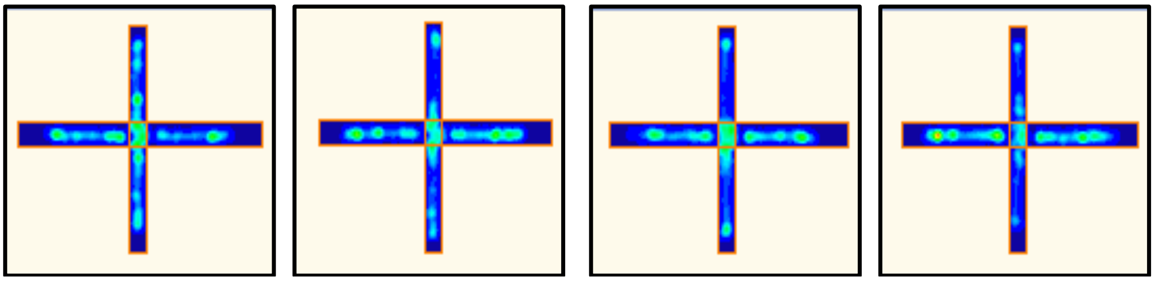

SD SD+BUT HP HP+BUT
